# Stem Cell Factor in Combination With Granulocyte Colony-Stimulating Factor Protects the Brain From Capillary Thrombosis-Induced Ischemic Neuron Loss in a Mouse Model of CADASIL

**DOI:** 10.3389/fcell.2020.627733

**Published:** 2021-01-12

**Authors:** Suning Ping, Xuecheng Qiu, Maria E. Gonzalez-Toledo, Xiaoyun Liu, Li-Ru Zhao

**Affiliations:** ^1^Department of Neurosurgery, State University of New York, Upstate Medical University, Syracuse, NY, United States; ^2^Department of Neurology, Cellular Biology and Anatomy, Louisiana State University Health Sciences Center, Shreveport, LA, United States

**Keywords:** CADASIL, SCF, G-CSF, capillary thrombosis, microinfarction

## Abstract

Cerebral autosomal dominant arteriopathy with subcortical infarct and leukoencephalopathy (CADASIL) is a Notch3 mutation-induced cerebral small vessel disease, leading to recurrent ischemic stroke and vascular dementia. There is currently no treatment that can stop or delay CADASIL progression. We have demonstrated the efficacy of treatment with combined stem cell factor (SCF) and granulocyte colony-stimulating factor (G-CSF) (SCF+G-CSF) in reducing cerebral small vessel thrombosis in a TgNotch3R90C mouse model of CADASIL. However, it remains unknown whether SCF+G-CSF treatment protects neurons from microvascular thrombosis-induced ischemic damage. Using bone marrow transplantation to track thrombosis, we observed that capillary thrombosis was widely distributed in the cortex, striatum and hippocampus of 22-month-old TgNotch3R90C mice. However, the capillary thrombosis mainly occurred in the cortex. Neuron loss was seen in the area next to the thrombotic capillaries, and severe neuron loss was found in the areas adjacent to the thrombotic capillaries with bifurcations. SCF+G-CSF repeated treatment significantly attenuated neuron loss in the areas next to the thrombotic capillaries in the cortex of the 22-month-old TgNotch3R90C mice. Neuron loss caused by capillary thrombosis in the cerebral cortex may play a crucial role in the pathogenesis of CADASIL. SCF+G-CSF treatment ameliorates the capillary thrombosis-induced ischemic neuron loss in TgNotch3R90C mice. This study provides new insight into the understanding of CADASIL progression and therapeutic potential of SCF+G-CSF in neuroprotection under microvascular ischemia in CADASIL.

## Introduction

Cerebral autosomal dominant arteriopathy with subcortical infarcts and leukoencephalopathy (CADASIL) is the most common monogenic cause of stroke and vascular dementia in adults (Chabriat et al., 2009). Currently, the pathogenesis of CADASIL remains poorly understood, and there is no treatment that can stop or delay CADASIL progression.

CADASIL is caused by mutations in the NOTCH3 gene (Joutel et al., 1996). NOTCH3-encoded Notch3 receptor is predominantly expressed in vascular smooth muscle cells (VSMCs) of small arteries (Wang et al., 2012) and pericytes of capillaries (Wang et al., 2014). Due to the specific distribution of Notch3, the typical pathologies in the brain are mainly found in small arteries and capillaries in both CADASIL patients and mouse models (Joutel, 2011). Pathological changes in endothelial cells (ECs) have been observed in CADASIL patients and the TgNotch3R90C mouse model of CADASIL (Ruchoux and Maurage, 1998; Ping et al., 2018, 2019). It has also been revealed that disrupted blood-brain barrier (BBB) integrity (Ping et al., 2018), increased thrombosis in cerebral small vessels (Ping et al., 2018), reduced cerebral blood vessel density (Liu et al., 2015; Ping et al., 2019) and impaired endothelium-dependent vasodilation (Stenborg et al., 2007) occur in CADASIL patients and TgNotch3R90C mice. Endothelial dysfunction is crucially involved in vascular ischemia as ECs shift from an anti-thrombotic to a pro-thrombotic stage when their function is dysregulated (Yau et al., 2015).

Cerebral microcirculation plays a vital role in brain health. Cerebral capillaries with a total length of ∼400 miles in humans are the primary site of oxygen, nutrient and metabolic exchange (Cipolla, 2009). Cerebral microcirculation impairment is a central pathology in Alzheimer’s dementia (Iadecola, 2015; van de Haar et al., 2015), while it remains unclear about the pathological role of cerebral microvascular impairment in CADASIL. There is a knowledge gap about the involvement of cerebral capillary thrombosis in ischemic neuronal death in CADASIL.

Stem cell factor (SCF) and granulocyte colony-stimulating factor (G-CSF) are the essential hematopoietic growth factors regulating blood cell production and bone marrow stem cell survival (Welte et al., 1985; Zsebo et al., 1990). Recently, we have demonstrated that SCF+G-CSF treatment ameliorates cerebrovascular endothelial cell (EC) damage and reduces cerebral capillary thrombotic formation in TgNotch3R90C mice (Ping et al., 2018, 2019). In addition, SCF+G-CSF treatment has been shown to reduce infarction size in acute ischemic stroke in a rat model of focal cerebral ischemia (Zhao et al., 2007b). However, whether SCF+G-CSF treatment could enhance neuronal survival in the area of thrombotic capillary remains elusive.

This study aims to determine the distribution of capillary thrombosis in the brain, the existence of cerebral capillary thrombosis-caused ischemic neuron loss, and the efficacy of SCF+G-CSF treatment in reducing microvascular ischemic damage in TgNotch3R90C mice.

## Materials and Methods

### Animals and Treatments

All procedures in this study were approved by Institutional Animal Care and Use Committee at SUNY Upstate Medical University and LSUHSC.

**FIGURE 1:**
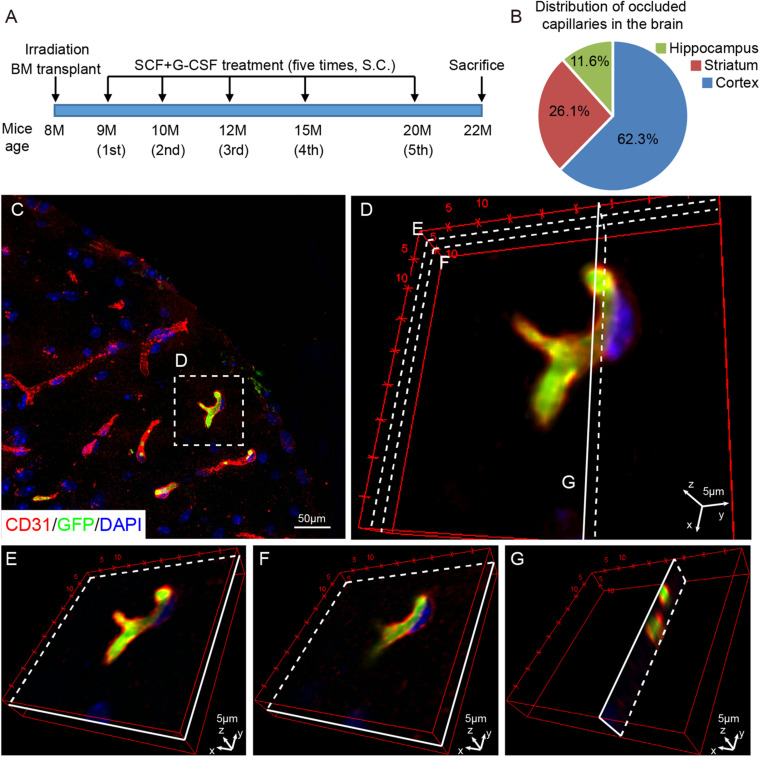
Capillary thrombosis is mainly located in the cortex of TgNotch3 mice. **(A)** Schematic diagram of the experiment. Eight-month-old TgNotch3R90C mice received lethal dose irradiation and bone marrow transplantation. At 9 months of age, mice received subcutaneous (s.c.) injections of SCF+G-CSF or vehicle solution for 5 days. The same treatment was repeatedly administered at the age of 10, 12, 15, and 20 months. All the mice were euthanized at the age of 22 months (*n* = 5/group). **(B)** A pie chart displays the percentage of thrombotic capillaries in the brain of 22-month-old TgNotch3R90C mice. The thrombotic capillaries were detected by GFP expressing blood cells that occluded capillaries. **(C)** Representative confocal image shows that GFP expressing blood cells occlude capillaries (<10 mm in diameter) in the cerebral cortex of a 22-month-old TgNotch3R90C mouse. **(D)** Three-dimensional image shows the capillary occluded by GFP expressing blood cells. Three different layers were selected to show that the capillary was completely occluded by GFP expressing blood cells. **(E,F)** Three-dimensional images illustrate that GFP expressing blood cells occlude a cerebral capillary at two different longitudinal layers [see the areas labeled with **(E,F)** in panel **D**]. **(G)** Three-dimensional image illustrates that GFP expressing blood cells occlude a cerebral capillary in a transverse view [see the area labeled with **(G)** in panel **D**]. Scale bars, 50 μm in panel **(C)** and 5 μm in panels **(D–G)**.

Transgenic mice (TgNotch3R90C) carrying a full-length human NOTCH3 gene with the Arginine-to-Cysteine (Arg90Cys) mutation driven by the SM22α promoter were used as a mouse model of CADASIL (Ruchoux et al., 2003). The original breeders were generously provided as gifts from Dr. Anne Joutel (Faculté de Médecine, Paris, France). Eight-month-old male TgNotch3R90C mice were randomly divided into two groups: a vehicle control group and an SCF+G-CSF-treated group. All mice received a lethal dose of radiation (900 rad) to destroy their own bone marrow. Within 24 h after irradiation, bone marrow isolated from transgenic mice ubiquitously expressing enhanced green fluorescent protein (GFP) (UBC-GFP mice) was transplanted to the irradiated mice. One month after bone marrow transplantation, the first treatment of SCF+G-CSF was initiated at 9 months of age, which is 1 month before cerebrovascular dysfunction is shown in TgNotch3R90C mice (Ruchoux et al., 2003; Lacombe et al., 2005). Recombinant mouse SCF (100 μg/kg, diluted with saline) (PeproTech, Rocky Hill, NJ, United States) and recombinant human G-CSF (50 μg/kg, diluted with 5% dextrose) (Amgen, Thousand Oaks, CA, United States) were subcutaneously administered for 5 days. An equal volume of vehicle solution (50% saline and 50% of 5% dextrose) was injected into the control mice. The same treatment was then repeated an additional 4 times at the ages of 10, 12, 15, and 20 months. The final treatment was given at 200 μg/kg of SCF and 50 μg/kg of G-CSF to further enhance the effectiveness. Mice were euthanized at the age of 22 months (*n* = 5/group) (Figure 1A).

### Bone Marrow Transplantation

The bone marrow of UBC-GFP mice (C57BL/6 background) was transplanted into the TgNotch3R90C mice (C57BL/6 background) to track blood clots (thrombosis) in the cerebral capillaries of TgNotch3R90C mice. UBC-GFP mice (male, 6 to 8 weeks old, Jackson Laboratory) were anesthetized with Avertin (0.4 g/kg body weight, intraperitoneally) (Sigma-Aldrich, St. Louis, MO, United States) and euthanized. The femur bones were dissected and placed into a cell culture dish with ice-cold sterile Hanks Balanced Salt Solution (HBSS) (ThermoFisher Scientific, Pittsburgh, PA, United States). Bone marrow cells were flushed out, gently triturated, and filtered through a 70 μm nylon mesh (Corning, Fisher Scientific, Pittsburgh, PA, United States). Harvested cells were centrifuged, re-suspended with HBSS into single cell suspension and then transplanted to the irradiated TgNotch3R90C mice by tail vein injection (1 × 10^7^ bone marrow cells in 0.6 ml HBSS per mouse).

### Brain Tissue Preparation and Immunohistochemistry

After being anesthetized with Avertin (0.4 g/kg, intraperitoneally), mice were euthanized by transcardiac perfusion of phosphate-buffered saline (PBS) (ThermoFisher Scientific, Pittsburgh, PA, United States) containing heparin (10 U/ml, Sagent Pharmaceuticals) followed by 10% neutral buffered formalin (Sigma-Aldrich, St. Louis, MO, United States). Brains were removed and post-fixed in the same fixative solution overnight at 4°C, and then dehydrated in 30% sucrose solution (Sigma-Aldrich, St. Louis, MO, United States) in PBS for 2 days at 4°C. Brains were sectioned at a thickness of 30 μm using a Cryostat (Leica Biosystems, Wetzlar, Germany). All brain sections were stored at −20°C with anti-freeze buffer containing 30% ethylene glycol and 30% glycerol (Sigma-Aldrich, St. Louis, MO, United States) prepared in PBS until used for immunohistochemistry.

Two brain sections per mouse (bregma 0.02 mm to −1.82 mm) were used for immunohistochemistry. After brain sections were rinsed with PBS, nonspecific binding was blocked with 10% donkey serum prepared in 1% bovine serum albumin (BSA, IgG free, Jackson ImmunoResearch Laboratories, West Grove, PA, United States) and 0.3% Triton X-100 (Sigma-Aldrich, St. Louis, MO, United States) solution for 1 h at room temperature. For the primary antibodies from mouse origin, brain sections were further blocked with mouse-on-mouse blocking reagent (M.O.M^TM^, Vectashield, Vector Laboratories, Burlingame, CA, United States). Brain sections were incubated with rat anti-mouse CD31 (1:50) (BD biosciences, San Jose, CA, United States), mouse anti-mouse NeuN (1:600) (ThermoFisher Scientific, Pittsburgh, PA, United States) and goat anti-mouse GFP (1:600) (Novus Biologicals, Littleton, CO, United States) primary antibodies at 4°C overnight. The next day, brain sections were washed with PBS and then incubated with the appropriate secondary antibody for 2 h at room temperature in the dark. The secondary antibodies used were Alexa Fluor 594-conjugated donkey anti-rat (1:500), Alexa Fluor 594-conjugated donkey anti-mouse (1:500) and Alexa Fluor 488-conjugated donkey anti-goat (1:500) (ThermoFisher Scientific, Pittsburgh, PA, United States). The antibodies were all diluted in PBS containing 1% BSA and 0.3% TritonX-100. Nuclei were stained with mounting medium (VECTASHIELD) containing DAPI (Vector Laboratories, Burlingame, CA, United States). Images were taken with a Zeiss 780 confocal microscope (Carl Zeiss Microscopy, LLC, Thornwood, NY, United States).

### Statistical Analysis

Data analysis was performed in a blinded manner. Two-group comparisons were analyzed using a student *t*-test based on the distribution of sample data. Two-way analysis of variance (ANOVA) followed by LSD *post hoc* multiple comparison tests were used to analyze two factor comparisons. All the data are presented as mean ± standard error of mean (SEM), and results were considered significantly different when a *p*-value was less than 0.05. Analyses were performed, and data were displayed using Prism software (GraphPad Software, Inc., La Jolla, CA, United States).

## Results

### Most Capillary Thrombosis in the Brains of TgNotch3R90C Mice Occurs in the Cortex

We first examined the distribution of the capillary thrombosis in the brains of TgNotch3R90C mice by detecting bone marrow-derived GFP positive blood cells that occluded the capillaries (capillaries: <10 μm in diameter) through immunofluorescence double staining of CD31 (EC marker) and GFP (bone marrow-derived blood cells). We observed that capillary thrombosis was randomly located in the brain. The most capillary thrombosis was seen in the cortex, which accounted for 62.3% of total occluded capillaries. The frequency of capillary thrombosis in the striatum and hippocampus was 26.1% and 11.6%, respectively, (Figure 1B). To further confirm that the capillaries were occluded by blood cells, three dimensional images were captured with a confocal microscope. We found that the occluded capillaries were filled with GFP positive bone marrow-derived blood cells (Figures 1C–G), indicating that capillary thrombosis occurs in the brains of TgNotch3R90C mice.

### SCF+G-CSF Treatment Attenuates Neuron Loss in the Areas Next to Thrombotic Capillaries in the Cortex of TgNotch3R90C Mice

**FIGURE 2:**
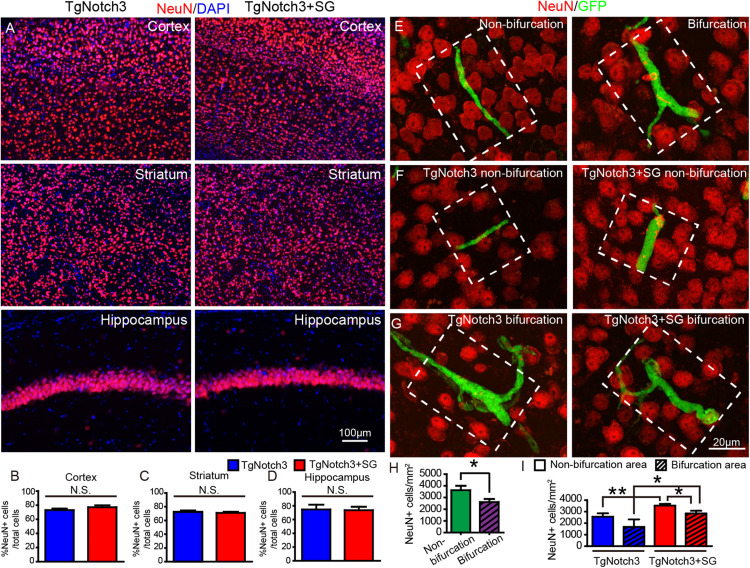
SCF+G-CSF treatment attenuates neuron loss in the areas next to the thrombotic capillaries in the cerebral cortex of TgNotch3R90C mice. Detection of neuron loss: NeuN positive neurons were quantified within the white dotted square area which is 50 μm adjacent to the occluded capillaries. **(A)** Representative confocal images show NeuN positive neurons in the cerebral cortex, striatum and hippocampus of TgNotch3R90C mice with or without SCF+G-CSF treatment. Red: NeuN positive neurons. Blue: DAPI nuclear counterstain. **(B–D)** Statistical analysis data show that the number of NeuN positive neurons in the cortex, striatum and hippocampus of 22-month-old TgNotch3R90C mice has no difference between vehicle controls and SCF+G-CSF-treated mice. **(E)** Representative confocal images show NeuN positive neurons in the microareas adjacent to the thrombotic capillaries with or without bifurcation. Red: NeuN positive cells. Green: GFP expressing blood cells that occlude capillaries. **(F)** Representative confocal images show NeuN positive neurons adjacent to the thrombotic capillaries without bifurcation in the cortex of TgNotch3R90C mice with or without SCF+G-CSF treatment. **(G)** Representative confocal images illustrate NeuN positive neurons next to the thrombotic capillaries with bifurcations in the cortex of TgNotch3R90C mice with or without SCF+G-CSF treatment. **(H)** Statistical analysis data show that the number of NeuN positive neurons in the areas next to the thrombotic capillaries with bifurcation is significantly decreased as compared with the areas next to the thrombotic capillaries without bifurcation in the cortex of all 22-month-old TgNotch3R90C mice including both SCF+G-CSF treated and non-treated mice. **(I)** Two-way ANOVA statistical analysis data show that SCF+G-CSF treatment significantly enhances neuron survival in both the areas adjacent to the thrombotic capillaries with and without bifurcation. The SCF+G-CSF-enhanced neuron survival is more robust in the area of thrombotic capillaries without bifurcation than in the area of thrombotic capillaries with bifurcation. *N* = 5. *p < 0.05, **p < 0.01.

Next, we sought to determine the involvement of capillary thrombosis in neuronal damage and the effect of SCF+G-CSF treatment in neuroprotection in the brains of TgNotch3R90C mice. First, we quantified the number of neurons in the cortex, striatum and hippocampus through NeuN immunostaining. We observed that the number of NeuN positive neurons was not significantly different between the SCF+G-CSF-treated and non-treated TgNotch3R90C mice in the cortex, striatum and hippocampus (Figures 2A–D). We then quantified the number of NeuN positive neurons in the microareas next to the occluded capillaries (the microarea = 50 μm from the center of occluded capillary × the length of occluded capillary) in the cerebral cortex of TgNotch3R90C mice (Figures 2E–I). We selected the cortex for determining capillary thrombosis-induced neuron damage due to its high frequency of capillary thrombosis. We found that the number of NeuN positive neurons was significantly decreased in the areas next to the thrombotic capillaries with bifurcation as compared to areas adjacent to the thrombotic capillaries without bifurcation (Figures 2E and H, *p* < 0.05). In the areas next to the thrombotic capillaries with non-bifurcation, SCF+G-CSF treatment significantly increased the number of NeuN positive neurons (Figures 2F and I, *p* < 0.01). Moreover, the number of NeuN positive neurons was also significantly increased in the areas adjacent to the thrombotic capillaries with the bifurcation after SCF+G-CSF treatment (Figures 2G and I, *p* < 0.05). However, SCF+G-CSF treatment-enhanced neuron survival was more robust in the microareas next to thrombotic capillaries without bifurcation than in the microareas next to thrombotic capillaries with bifurcation (Figure 2I, *p* < 0.05). These data indicate that the structure of cortical capillaries may influence the microenvironment in the brains of the TgNotch3R90C mice, which affects the severity of capillary thrombosis-caused ischemic neuron damage. The findings also demonstrate that SCF+G-CSF treatment protects neurons from ischemic injury caused by capillary thrombosis in the cortex of TgNotch3R90C mice. The extent of SCF+G-CSF-enhanced neuron survival in the microareas next to the thrombotic capillaries is also influenced by the structure of the thrombotic capillary.

## Discussion

**FIGURE 3:**
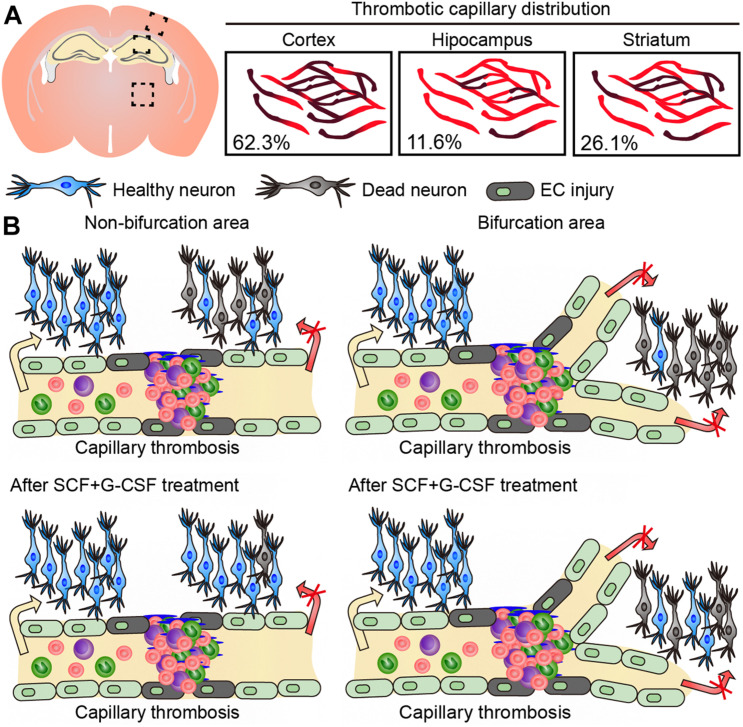
Graphic summary of the findings in this study. **(A)** The majority of capillary thrombosis is seen in the cerebral cortex of 22-month-old TgNotch3R90C mice. **(B)** Neuron loss occurs in the area next to thrombotic capillaries. The neuron loss becomes more severe in the areas of thrombotic capillaries with bifurcations. SCF+G-CSF treatment attenuates the neuron loss adjacent to the thrombotic capillaries in the cerebral cortex of 22-month-old TgNotch3R90C mice.

In the present study, we have identified that the majority of capillary thrombosis in the brain of TgNotch3R90C mice occurs in the cortex. We have also, for the first time, demonstrated that (1) microscopically detectable neuron loss exists in the area next to the thrombotic capillary, (2) the neuron loss becomes more severe in the areas adjacent to the thrombotic capillaries with bifurcations, and (3) SCF+G-CSF treatment ameliorates the neuron loss adjacent to the thrombotic capillaries in the cerebral cortex of 22-month-old TgNotch3R90C mice (Figure 3).

We employed the bone marrow transplantation in this study to track blood cells in the cerebral capillary thrombosis. Before the bone marrow transplantation, all the mice received a lethal dose (900 rad) of radiation to ablate their bone marrow. This bone marrow transplantation approach has been successfully used for tracking bone marrow-derived cells in our previous studies (Piao et al., 2009; Li et al., 2011; Liu et al., 2015). It has been well documented that transplantation of a genetically identical graft (syngeneic graft or autologous bone marrow) does not lead to rejection (Duran-Struuck and Dysko, 2009). The UBC-GFP mice (bone marrow donors) and TgNotch3R90C mice (bone marrow recipients) both have C57BL/6 genetic background. The bone marrow transplantation was performed within 24 h after irradiation. In the present study, we did not observe bone marrow failure or the graft-versus-host disease in TgNotch3R90C mice. The commonly used C57BL/6 mice have been shown to tolerate the radiation doses of 1,000 to 1,100 rad (Duran-Struuck et al., 2008). In the current study, we did not see irradiation-induced injury in TgNotch3R90C mice. Taken together, all the TgNotch3R90C mice used in this study were in good condition after irradiation and bone marrow transplantation.

### Capillary Thrombosis Leads to Local Neuron Damage in the Brains of TgNotch3R90C Mice

Our data show that capillary thrombosis is widely distributed in the cerebral cortex, striatum and hippocampus of 22-month-old TgNotch3R90C mice. These findings are consistent with our earlier study (Ping et al., 2018) revealing EC degeneration/damage-induced cerebral capillary thrombosis in 22-month-old TgNotch3R90C mice. These observations suggest that cerebral capillary thrombosis is involved in the pathogenesis of CADASIL. It has been questioned whether thrombosis or hemorrhage in cerebral small vessels leads to CADASIL-caused lacunar infarcts because microbleeds are observed in CADASIL patient’s brain but there is a lack of evidence that supports thrombotic genesis of ischemic events (Di Donato et al., 2017). Here we have provided key evidence revealing that capillary thrombosis occurs in the brains of mice carrying CADASIL-related Notch3 mutations and that the capillary thrombosis leads to ischemic neuron loss.

In TgNotch3R90C mice, degenerating ECs and impaired cerebrovascular function are found as early as 10 months of age (Ruchoux et al., 2003; Lacombe et al., 2005). Our previous study has shown EC damage and thrombosis in the cerebral capillaries and small vessels of 22-month-old TgNotch3R90C mice (Ping et al., 2018), suggesting that endothelial dysfunction-induced thrombosis occurs in the cerebral microvessels because ECs shift from an anti-thrombotic to a prothrombotic stage when their function is dysregulated (Yau et al., 2015). The underlying mechanism of EC degeneration/damage in CADASIL remains elusive. It is well documented that VSMCs and pericytes are required for maintaining vascular function and stabilization (Franco et al., 2011; Miyagawa et al., 2019). Pharmacological or genetic ablation of pericytes leads to reduced EC survival, BBB breakdown and vascular degeneration (Franco et al., 2011; Winkler et al., 2011). In our earlier studies, we have observed the autophagic degeneration of capillary pericytes and capillary EC degeneration/damage in the cerebral cortex of 22-month-old TgNotch3R90C mice (Gu et al., 2012; Ping et al., 2018), suggesting that capillary EC degeneration/damage in the brains of 22-month-old TgNotch3R90C mice is associated with capillary pericyte degeneration.

Our data have also revealed that the majority of blood-occluded capillaries are located in the cerebral cortex, and that neuron loss occurs in the areas next to the thrombotic capillaries in TgNotch3R90C mice. These observations are in line with clinical findings revealing microinfarcts in the cortex of CADASIL patients (Jouvent et al., 2011; De Guio et al., 2014). These findings renew the traditional notion that CADASIL only affects the subcortical white matters (Dichgans, 2002), and provide insightful evidence demonstrating that capillary thrombosis-caused ischemic damage in the cerebral cortex may play a crucial role in the pathogenesis of CADASIL.

It remains unclear why the capillary thrombosis is mainly located in the cortex, instead of the striatum and hippocampus. It has been documented that the topological distribution of the capillary network is different throughout the brain to match the regional metabolism (Shaw et al., 2019; Zhang et al., 2019). The vascular network shows distinct distribution and density in the cortex, hippocampus and striatum. The cortical parenchyma is full of abundant vessels, which are well arranged into a mesh-like network. The vascular density in the hippocampus is significantly lower than the cortical vessel density (Zhang et al., 2019). No obvious difference of vascular density is seen between the striatum and cortex (Di Giovanna et al., 2018). Based on these studies, we postulate that the different varieties of metabolic rates with the different distribution and density of vascular network in different brain regions may influence the formation of capillary thrombosis in the TgNotch3R90C mice.

Vascular occlusion and hemorrhage are the vascular events that lead to regional neuronal death (i.e., infarcts) (Nishimura and Schaffer, 2013). Here we have observed that blood clots completely occlude the capillaries, demonstrating the formation of capillary occlusion (i.e., capillary thrombosis). The occlusion of capillaries prevents blood flow from reaching a small territory of brain tissue leading to ischemic damage and local neuron death. Our findings have also revealed that neuron loss is increased in the microareas next to the occluded capillaries with bifurcations as compared to the microareas adjacent to the occluded capillaries without bifurcations, indicating that the severity of ischemia-induced neuron loss is capillary structure-related. In the cerebral vasculature, the number of vascular branches is dramatically increased in the capillaries as compared with other types of blood vessels (Reina-De La Torre et al., 1998). The bifurcation is the major junction in the capillary bed (Gould et al., 2017). Blood flow starts from the arterial inlet and makes random choices at each bifurcation, making the blood flow complex at the site of the bifurcation; as a result, it leads to the ECs in the bifurcation area being more vulnerable to damage in the presence of risk factors for vascular diseases (De Syo et al., 2005). It is possible that once the thrombotic occlusions occur at the bifurcation site of the capillary network, two or more downstream capillaries will be affected, which leads to an increased severity of ischemia and neuronal loss in the territory of affected capillaries.

### SCF+G-CSF Treatment Ameliorates Ischemic Neuron Loss in the Area Next to the Thrombotic Capillary

In the present study, SCF+G-CSF treatment-reduced neuronal loss has been observed in the areas adjacent to the thrombotic capillaries, demonstrating the neuroprotective efficacy of SCF+G-CSF on capillary thrombosis-induced ischemic damage in the brains of TgNotch3R90C mice. The effects of SCF and G-CSF in neuroprotection have been reported in focal cerebral ischemia. Focal cerebral ischemia is produced by occlusion of a relatively large artery, the middle cerebral artery (MCA), leading to massive brain tissue loss (i.e., infarct) in the territory of the occluded MCA. In rodent models of focal cerebral ischemia, systemic administration of SCF (Zhao et al., 2007b), G-CSF (Schneider et al., 2005; Zhao et al., 2007b), and SCF+G-CSF (Kawada et al., 2006; Zhao et al., 2007b) initiated in the acute phase of ischemic stroke (<48 h after MCA occlusion) results in the reduction of infarction size. Strikingly, subcutaneous injection of SCF+G-CSF in the subacute phase (during 11–20 days post-ischemia) (Kawada et al., 2006) or in the chronic phase (14 weeks post-ischemia) (Zhao et al., 2007a) of ischemic stroke still shows significant reductions of infarct volume. These studies have demonstrated the effectiveness of SCF+G-CSF treatment in protecting neurons from both acute ischemic damage and the focal cerebral ischemia-induced long-term progressive neuron loss. In contrast to stroke that occurs as a sudden event, CADASIL causes progressive damage in cerebral small arteries and capillaries, leading to progressive thrombosis in these vessels. Most likely, when we give treatment to different ages of TgNotch3R90C mice, the capillary thrombosis-induced ischemic neuron loss at different locations may be under different post-ischemic stages/phases. SCF+G-CSF treatment may exert its universal protection against neuron death from the different stages/phases.

The mechanism underlying SCF+G-CSF-enhanced neuroprotection in different phases of cerebral ischemia remains unclear. Expression of receptors for SCF (Zhao et al., 2007b) and G-CSF (Schneider et al., 2005) on neurons reflects their direct efficacy in neuroprotection. In cultured primary cortical neurons, SCF protects neurons from excitotoxicity through MEK/ERK and PI3K/AKT/NF-kB pathways and from apoptosis through PI3K/AKT/NF-kB/Bcl-2 signaling, and the SCF-enhanced neuroprotection is dependent on its receptor (c-kit) expression (Dhandapani et al., 2005). G-CSF receptor, G-CSFR, is robustly expressed in the peri-infarct neurons of rat brain (Schneider et al., 2005) and human brain (Hasselblatt et al., 2007) in the acute phase of ischemic stroke. G-CSF counteracts programmed neuron death via PI3K mediation in cultured cortical neurons (Schneider et al., 2005).

In addition to the direct effects of SCF and G-CSF in neuroprotection demonstrated in *in vitro* studies, many *in vivo* studies have revealed that SCF and G-CSF may protect the brain from post-ischemic neuron loss through an indirect way. It has been shown that brain ischemia-triggered neuroinflammation in both the acute and subacute phases leads to secondary neuron loss including apoptotic neuron death (Duris et al., 2018; Jayaraj et al., 2019). G-CSF treatment in the acute phase of focal cerebral ischemia suppresses pro-inflammatory cytokines and inflammatory mediators in the peri-ischemic areas (Gibson et al., 2005; Sehara et al., 2007), reduces the disruption of the BBB (Lee et al., 2005), inhibits peripheral inflammatory cell infiltration to the ischemic hemisphere (Lee et al., 2005) and reduces neuronal apoptosis in the ipsilesional cortex (Solaroglu et al., 2006). SCF+G-CSF treatment in the subacute phase of focal cerebral ischemia upregulates IL-10, an anti-inflammatory cytokine, in the ipsilesional cortex (Morita et al., 2007). In our earlier study, the level of vascular endothelial growth factor (VEGF) is decreased in the brains of TgNotch3R90C mice, while SCF+G-CSF treatment increases cerebral VEGF in TgNotch3R90C mice (Ping et al., 2019). Intracerebral injection of VEGF reduces infarct volume in focal cerebral ischemia, and the direct effect of VEGF in neuroprotection is also demonstrated in *in vitro* hypoxia models (Greenberg and Jin, 2013). It would be possible that increased cerebral VEGF may also contribute to the SCF+G-CSF-induced neuroprotection in the area of capillary thrombosis in TgNotch3R90C mice.

Our previous studies have demonstrated that repeated treatments with SCF+G-CSF reduce cerebral capillary thrombosis, attenuate capillary thrombosis in the bifurcation regions (Ping et al., 2018), enhance angiogenesis and increase blood vessel density in the brains of TgNotch3R90C mice (Liu et al., 2015; Ping et al., 2019), which may lead to increasing collateral circulation and ameliorating ischemic damage in the areas next to the thrombotic capillaries. Our present findings reveal that SCF+G-CSF-reduced neuron loss is more robust in the areas with a single thrombotic capillary than the areas with multiple thrombotic capillaries (i.e., thrombotic capillaries with bifurcations). These findings suggest that the increase of collateral circulation by SCF+G-CSF may be more sufficient to rescue neurons from the microareas without severe ischemic damage than from the microareas with severe ischemic damage due to having multiple thrombotic capillaries.

In conclusion, this study provides new and important evidence demonstrating that capillary thrombosis-caused microvascular ischemic damage exists in the brains of 22-month-old TgNotch3R90C mice. The thrombotic capillaries are mainly located in the cortex, and the capillary thrombosis leads to local neuron loss. Systematic administration of SCF+G-CSF attenuates neuron loss in the areas next to the thrombotic capillaries. These findings advance toward an understanding of the pathological role of cortical capillary thrombosis-caused microvascular ischemia in the pathogenesis of CADASIL and reveal a new therapeutic target for developing treatment to ameliorate microvascular ischemia in CADASIL.

## Data Availability Statement

The original contributions presented in the study are included in the article, and further inquiries can be directed to the corresponding author.

## Ethics Statement

The animal study was reviewed and approved by the Institutional Animal Care and Use Committee at SUNY Upstate Medical University and LSUHSC.

## Author Contributions

SP, MG-T, and XL performed the experiment. SP prepared the first draft of the manuscript. XQ provided assistance in the data analysis. LRZ conceived the study, supervised the experiment, and revised the manuscript. All authors reviewed and approved the submitted version of the manuscript.

## Conflict of Interest

The authors declare that the research was conducted in the absence of any commercial or financial relationships that could be construed as a potential conflict of interest.
